# Vicious LQT induced by a combination of factors different from hERG inhibition

**DOI:** 10.3389/fphar.2022.930831

**Published:** 2022-07-22

**Authors:** Xinping Xu, Yue Yin, Dayan Li, Binwei Yao, Li Zhao, Haoyu Wang, Hui Wang, Ji Dong, Jing Zhang, Ruiyun Peng

**Affiliations:** Beijing Institute of Radiation Medicine, Beijing, China

**Keywords:** cisapride, QT prolongation, optical mapping, TDP, triangulation, dispersion, instability

## Abstract

Clinically, drug-induced torsades de pointes (TdP) are rare events, whereas the reduction of the human ether-à-go-go-related gene (hERG) current is common. In this study, we aimed to explore the specific factors that contribute to the deterioration of hERG inhibition into malignant ventricular arrhythmias. Cisapride, a drug removed from the market because it caused long QT (LQT) syndrome and torsade de pointes (TdP), was used to induce hERG inhibition. The effects of cisapride on the hERG current were evaluated using a whole-cell patch clamp. Based on the dose-response curve of cisapride, models of its effects at different doses (10, 100, and 1,000 nM) on guinea pig heart *in vitro* were established. The effects of cisapride on electrocardiogram (ECG) signals and QT interval changes in the guinea pigs were then comprehensively evaluated by multi-channel electrical mapping and high-resolution fluorescence mapping, and changes in the action potential were simultaneously detected. Cisapride dose-dependently inhibited the hERG current with a half inhibitory concentration (IC50) of 32.63 ± 3.71 nM. The complete hERG suppression by a high dose of cisapride (1,000 nM) prolonged the action potential duration (APD), but not early after depolarizations (EADs) and TdP occurred. With 1 μM cisapride and lower Mg^2+^/K^+^, the APD exhibited triangulation, dispersion, and instability. VT was induced in two of 12 guinea pig hearts. Furthermore, the combined administration of isoproterenol was not therapeutic and increased susceptibility to ventricular fibrillation (VF) development. hERG inhibition alone led to QT and ERP prolongation and exerted an anti-arrhythmic effect. However, after the combination with low concentrations of magnesium and potassium, the prolonged action potential became unstable, triangular, and dispersed, and VT was easy to induce. The combination of catecholamines shortened the APD, but triangulation and dispersion still existed. At this time, VF was easily induced and sustained.

## Introduction

Drug-induced long QT syndrome (LQT) is generally considered to be proarrhythmic and is often associated with torsade de pointes (TdP) arrhythmias and sudden death (De Bruin et al., 2005; Sanguinetti and Mitcheson, 2005; Hoffmann and Warner, 2006; Vandenberg et al., 2012). Human ether-à-go-go-related gene (hERG) blockade can cause LQT, and many drugs have been identified to pose a risk for arrhythmia because of hERG inhibition and prolongation of the action potential duration (APD) (De Bruin. et al., 2005; Sanguinetti and Mitcheson, 2005; Gintant, et al., 2006; Dennis, et al., 2012). Previous evidence supports this correlation between anti-hERG activity and pro-arrhythmic risk (De Bruin. et al., 2005). In fact, many drugs, such as cetirizine, loratadine, and ranolazine (Shenasa. et al., 2016) which cause QT and APD prolongation or hERG inhibition, are non-proarrhythmic. Since these typical cases still need case-by-case investigations, it is inaccurate to use QT prolongation and hERG inhibition to determine the pro-arrhythmic risk of drugs (Hondeghem, 2008; Friedman. et al., 2021). Meanwhile, hERG inhibition has a beneficial class III anti-arrhythmic effect. Consequently, other factors which differentiate hERG inhibition as benign and vicious should be taken into consideration but not hERG inhibition per se.

Cisapride, a gastrointestinal motility drug, has been removed from the market because of causing LQT and inducing TdP (Wysowski and Bacsanyi, 1996; Drolet. et al., 1998; Di Diego, et al., 2003a). Clinical evidence suggests that TdP occurs in approximately 1.5% of the patients taking cisapride. Significantly, however, patients who developed arrhythmia events induced by cisapride generally had a history of heart disease (e.g., atrial fibrillation, coronary heart disease, etc.), electrolyte disturbances, renal insufficiency, or long-term use of the drug in combination with other medicines that may cause arrhythmias or prolong QT intervals (Wysowski. and Bacsanyi, 1996). Hence, we hypothesized that in normal hearts, QT prolongation induced by hERG inhibition, such as that caused by cisapride alone does not induce TdP and that pathogenic changes in QT or the action potential may occur because of the action of certain other factors.

Taking cisapride as an example, in our present study, we investigated the changes in the action potential, especially changes in the ventricular repolarization, in isolated guinea pig hearts after treatment with cisapride alone or in combination with the action of other factors. The action potential or ventricular repolarization can be described precisely by its APD triangulation, instability, and dispersion. Optical mapping can be applied for the visual detection of changes in the ventricular action potential, which can provide an important evaluation index for the cardiac safety evaluation of drugs (Niu, et al., 2021; Wang. et al., 2021).

## Materials and methods

### Experimental animals

Guinea pigs (weighing 200–250 g, male or female) were purchased from SPF (Beijing) Biotechnology Co., Ltd. Each cage contained five of these animals fed normally in an SPF-grade animal facility, with *ad libitum* access to food and water. They were kept at 20–25°C, under good ventilation and a humidity of 50% ± 5%. The ethics approval number of this study is SGLL220305018 (Henan, Henan Province, China).

### Drug preparation

Cisapride powder (Sigma, United States) was dissolved in a DMSO solution to prepare a 10 μM stock solution, which was stored at −20°C for 2–4 weeks until use. The working solution was diluted with extracellular or KH solutions with a final DMSO concentration of less than 3‰.

### Cell culture

In this study, HEK293 cells stably expressing hERG were provided by Henan Scope Research Institute of Electrophysiology. The cells were cultured in a complete medium (DMEM medium plus 10% fetal bovine serum) in an incubator at 37°C and 5% CO_2_. After 2–3 days of adherent cell growth, when the cell density reached approximately 90% of the culture flask, the cells were digested with trypsin digestion solution for 1–2 min, gently blown, and centrifuged. Then, 2–3 ml of DMEM complete culture medium was used to disperse the cell precipitate. A volume of 20–50 μl of the mixture was next taken and placed on a sterile slide of a prepared Petri dish. Furthermore, 1 ml of the mixture was placed in the cell incubator for patch-clamp recording.

### Patch-clamp testing

The channel current was recorded in HEK293 cells stably expressing the hERG channel protein by the voltage-clamp technique with an Axon 700A patch-clamp amplifier (Molecular Devices, San Jose, CA, United States). The microelectrode was made of borosilicate hard glass blank, and the glass electrode was drawn by a puller. The following extracellular solution component concentrations (mM) in the K^+^ channel were used: NaCl 140, KCl 5.4, MgCl_2_ 1, CaCl_2_ 2, glucose 10, and HEPES 10, and the pH was adjusted to 7.4 with NaOH. The intracellular solution component concentrations (mM) used were KCl 140, Mg-ATP 4, MgCl_2_ 1, EGTA 5, and HEPES 10, and the pH was adjusted to 7.2 with KOH. All reagents were purchased from Sigma (Budapest, Hungary). The electrode resistance was 2–4 MΩ after filling the electrode solution. In the experiment, the slides pre-plated with cells were placed in a bath, single cells were selected, and a clear field of view was obtained by adjustment. A microelectrode manipulator was used to fill the electrode into the liquid for liquid potential compensation. The electrode was given negative pressure after contacting the cells. After the negative pressure was maintained for several seconds, the electrode tip formed a GΩ sealing connection with the surface of the cell membrane. Then, to compensate for the electrode capacitance, the negative pressure was applied to suck up the cell membrane. The series resistance and cell membrane capacitance were compensated. The electrophysiological recording was further performed at room temperature. The sampling rate was 10 kHz and the rate of Bessel filtering was 6 kHz. The experimental process was controlled by pCLAMP10 software and Axoclamp700A software equipped with an amplifier. The digital-to-analog converter completed the generation of stimulation signals and fed back the acquisition of signals.

### Optical mapping

Guinea pigs were intraperitoneally injected with heparin (3125 U/kg) and sacrificed approximately 15 min later (after isoflurane gas anesthesia). Inverted T-shaped thoracotomy was performed, and the heart was placed in a glass Petri dish filled with precooled bench-top solution. Then, the aorta was quickly found, and the excess tissue was cut off. Next, the aorta was carefully sleeved at the bottom of the cardiac cannula using forceps, tied with surgical sutures, and pre-prepared KH solution in a syringe was gently pushed into the heart to pump out the residual blood of the heart, followed by Langendorff perfusion at a perfusion rate of 8 ml/min and a perfusion temperature of 37 ± 0.5°C. A KH solution was used as the perfusion solution, which contained the following compounds (mM): NaCl 119, KCl 4, CaCl_2_ 1.8, MgCl_2_ 1, NaH_2_PO_4_ 1.2, NaHCO_3_ 25, and D-Glucose 10. The lower Mg^2+^/K^+^ perfusion solution contained 2 mM KCl and 0.5 mM MgCl_2,_ with the other components unchanged. The experiment was performed after the heart returned to a normal rhythm and remained stabilized for 15 min. Then, 100 ml of KH solution was added to a circulating perfusion tank, 300 μl of 1 mg/ml Blebbistatin (Abcam, United Kingdom) was added to the dosing port to arrest the heart, and 50 μl of Pluronic F127 (Invitrogen, United States) was added to the circulating perfusion tank for 10 min. Later, 100 μl of 1 mg/ml voltage-sensitive dye RH237 (Santa Cruz Biotechnology, United States) was successively added to the dosing port and circulated for 15 min. The dye-loaded heart was then moved to the imaging perfusion chamber, and a stimulating electrode was inserted at the apex for pacing stimulation. The ECG electrodes were attached to the RA and LV of the heart, respectively. The action potential of normal hearts was recorded using the OMS-PICE-2002 system (MappingLab, United Kingdom) with EMapRecord 5.0 (MappingLab, United Kingdom). Cisapride was then perfused sequentially at 10 nM, 100 nM, and 1,000 nM, and the drug effect was measured 10 min after drug perfusion. Data analysis was performed using a commercially available analysis program (EMapScope5.7, MappingLab, United Kingdom). The activation time was presented as an iso-chronogram (Lee, et al., 2012; Liao, et al., 2020).

### Statistical analyses

All patch-clamp recorded data were analyzed using Clampfit 10.6 (Molecular Devices, United States), OriginPro 8.0 (Origin Lab, United States), and Adobe Illustrator 10 (Adobe, United States). The concentration-response curve was fitted by the logistic equation *y* = *A*_2_+(*A*_1_-*A*_2_)/(1+(*x*/*x*_0_)^*p*^), where *x* is the drug concentration and *p* is the Hill coefficient. All data were expressed as means ± SEM. One-way ANOVA, followed by a multiple-comparison test, was used to evaluate multiple test treatments. A value of *p* < 0.05 was considered to indicate statistically significant differences. IC_50_ denotes the concentration determined for half-maximal inhibitory effects. In the figures, the designations for the *p-*values are: *p < 0.05, **p < 0.01, and ***p < 0.001, respectively.

## Results

### Dose-dependence of cisapride effect on the hERG current and overall cardiac action potential duration

**FIGURE 1:**
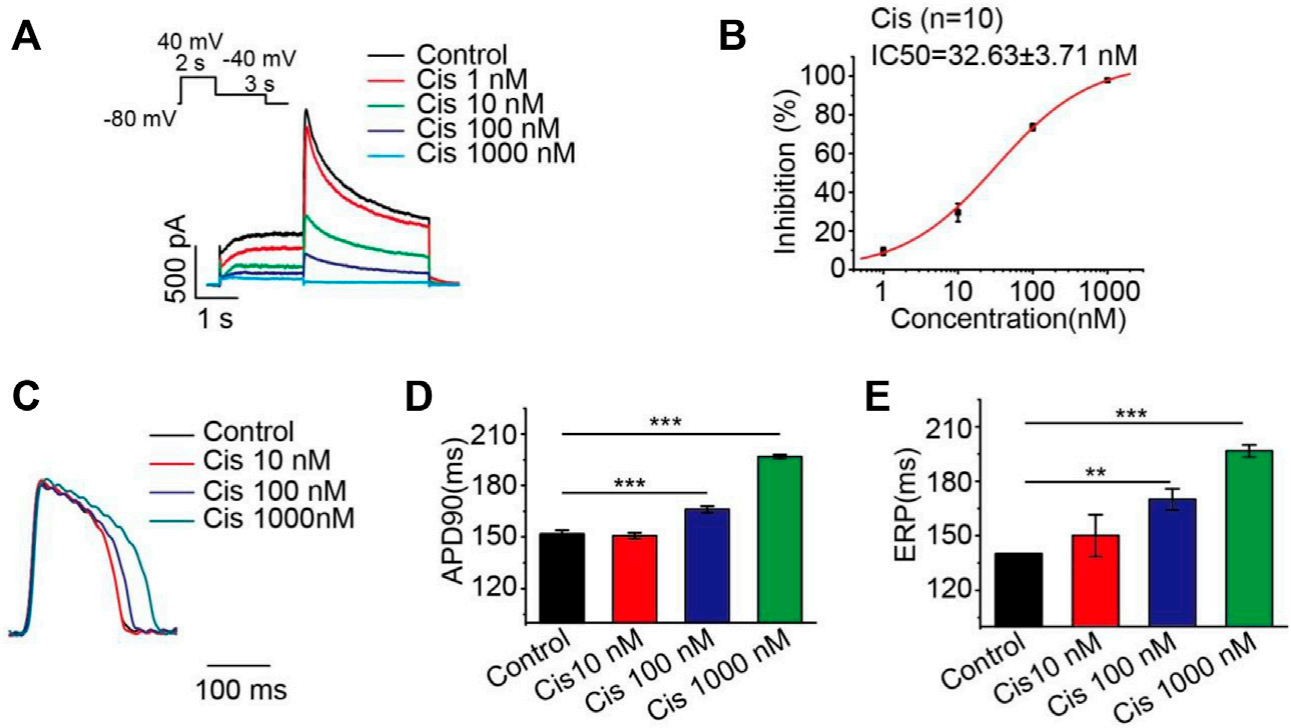
Cisapride dose-dependently inhibited the hERG current and increased APD and ERP. **(A)** Representative diagram of the concentration-dependent inhibition of the hERG channel by cisapride; **(B)** dose-response curve for the inhibition of the hERG channel by cisapride (*n* = 10); **(C)** representative graph of cisapride concentration-dependently increase APD; **(D)** APD and **(E)** ERP in the absence of different concentrations of cisapride (10, 100, and 1,000 nM) (*n* = 8).

Many studies have shown that cisapride-induced QT prolongation may be related to the block of potassium current, which prolongs the action potential repolarization in cardiomyocytes (Qian and Guo. 2010; Liang, et al., 2013). In this experiment, HEK293 cells stably expressing the hERG protein were studied, and it was found that cisapride dose-dependently inhibited the hERG current with an IC_50_ of 32.63 ± 3.71 nM (Figure 1B), and a high dose (1,000 nM) caused 100% inhibition of the hERG current (Figure 1A). Action potentials were recorded at a pacing rate of 4 Hz. In the whole heart, the maximum APD prolongation was achieved at 1,000 nM (Figures 1C,D), with no significant difference between 1,000 nM and the increased concentration of 3,000 nM (not displayed). Ventricular effective refractory period (ERP) analysis of the recorded results revealed that cisapride could dose-dependently increase the ERP, similar to APD90 (Figure 1E). However, based on these data alone, it is hard to discriminate whether cisapride has an arrhythmia-inducing effect.

### Arrhythmias (EAD/DAD/VT/VF) induced by high-frequency stimulation (50 Hz)

**FIGURE 2:**
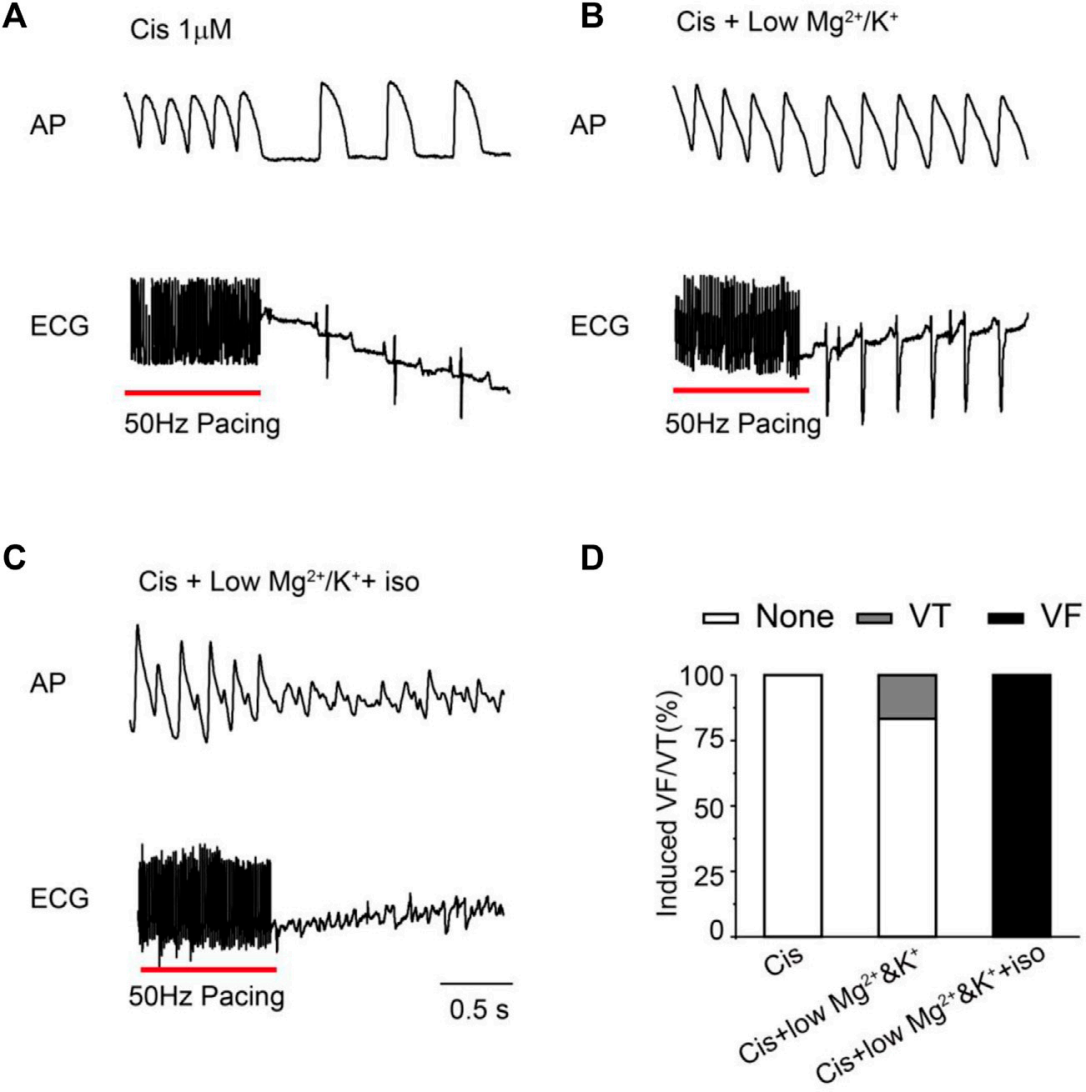
Representative traces and statistical graph of induced VF/VT under different conditions. **(A)** High-frequency stimulation induced VT/VF with 1 μM Cis (*n* = 18); **(B)** VT/VF was induced by high-frequency stimulation of 1 μM Cis + low Mg^2+^/K^+^(*n* = 12); **(C)** high-frequency stimulation induced VT/VF with 1 μM Cis + low Mg^2+^/K^+^+ iso (*n* = 7); **(D)** statistical chart of VT/VF induction rate under different conditions.

To further investigate the pro-arrhythmic risk of cisapride, we attempted to induce arrhythmias (EAD/DAD/VT/VF) with high-frequency stimulation (50 Hz) under different conditions (cisapride 1 μM alone, combined with lower Mg^2+^/K^+^ or continued with isoproterenol 0.5 μM) in Langendorff-perfusion guinea pig hearts. Using 1 μM cisapride alone hardly induced VT/VF by high-frequency stimulation (*n* = 18, Figure 2A). With the combination of 1 μM cisapride and lower Mg^2+^/K^+^, 2 of 12 guinea pig hearts induced VT but was not sustained and was reversible (Figure 2B). Furthermore, VF was observed in 100% of the hearts of the guinea pigs treated with 1 μM cisapride in combination with lower Mg^2+^/K^+^ and 0.5 μM isoproterenol. All VFs induced were continuous and irreversible (*n* = 7, Figure 2C). The probability of induced VF/VT was compared under different conditions (Figure 2D). The results showed that using cisapride 1 μM alone induced no VT/VF, whereas its combination with lower Mg^2+^/K^+^ or continued with 0.5 μM isoproterenol had different levels of pro-arrhythmic risk.

### Triangulation and dispersion changes after cisapride treatment or other factors added

**FIGURE 3:**
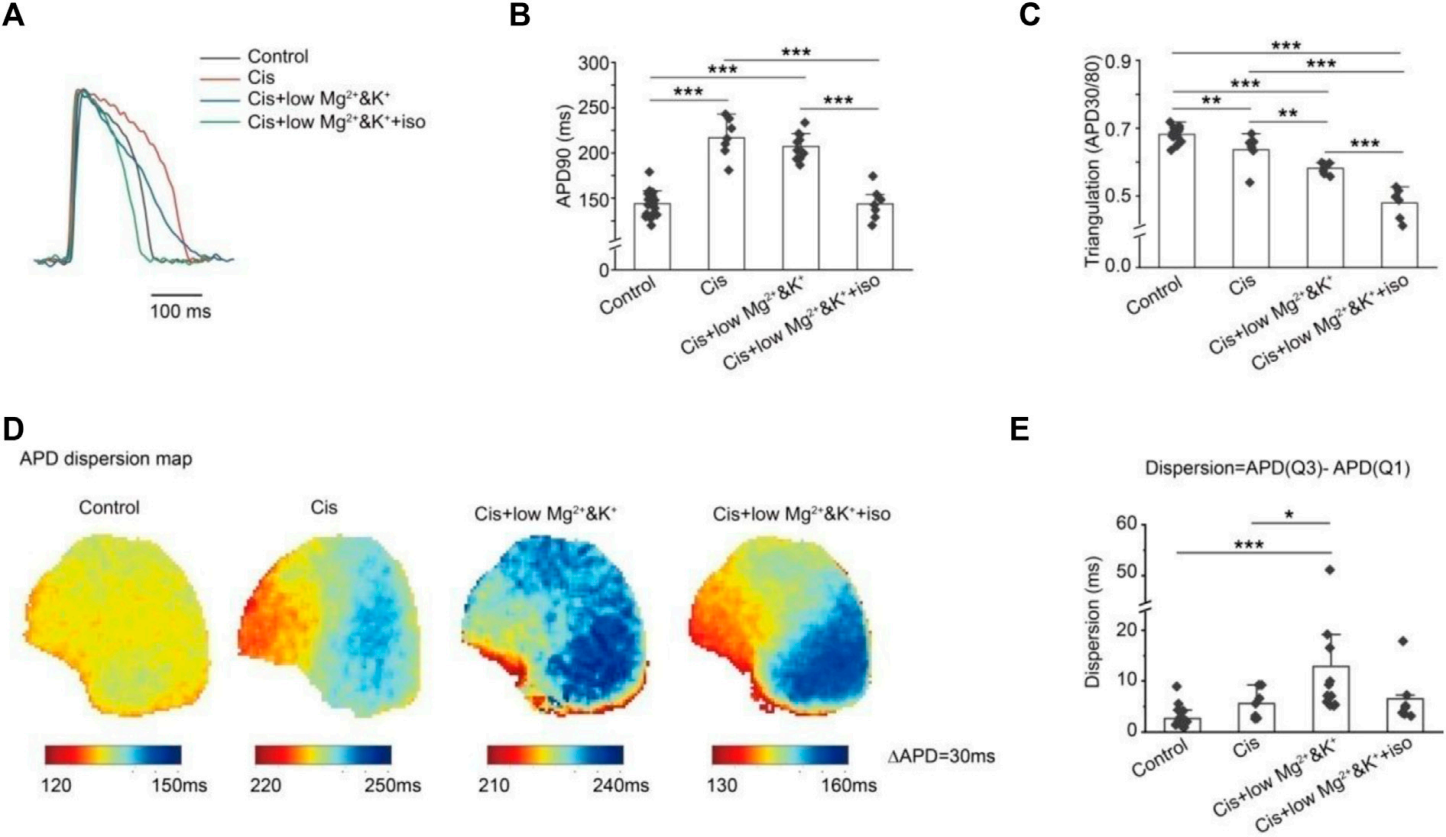
Effects of different conditions on AP morphology and dispersion. **(A)** Effects of different conditions on AP morphology; **(B)** quantitative statistics of APD prolongation under different conditions; **(C)** quantitative statistics of AP triangulation under different conditions. The APD30/APD80 ratio was used to quantify the AP triangular morphology under different conditions. A smaller ratio was associated with more significant AP triangular morphology; **(D)** optical mapping of ventricular AP under different conditions; **(E)** quantitative statistics of ventricular AP dispersion under different conditions were expressed by APD (Q3)-APD (Q1). We found that under low-magnesium and -potassium conditions, the dispersion of AP was significantly increased by cisapride (*p < 0.05; **p < 0.01; and ***p < 0.001).

We compared the graphics of the action potentials under different conditions (Figure3A). The action potential duration was significantly prolonged at 1 μM cisapride alone or its combination with lower Mg^2+^/K^+^, but it was shortened after isoproterenol was continued (Figure 3B). The APD30/APD80 ratio was used to represent the trend of triangulation. A smaller ratio indicated increased triangulation AP morphology. We found that the triangulation was more significant when 1 μM cisapride was given or combined with lower Mg^2+^/K^+^ or the combination was continued with isoproterenol (Figure 3C). Meanwhile, we compared the APD90 dispersion map of the anterior ventricular wall in Langendorff-perfusion guinea pig hearts (Figure 3D) and conducted a statistical analysis on its dispersion (Q3-Q1). We established that the dispersion of cisapride alone did not increase significantly compared with that in the control group, but the dispersion increased dramatically under the condition of lower Mg^2+^/K^+^(Figure 3E). Elevated dispersion might be a major risk of arrhythmias.

### Instability changes after cisapride treatment or other factors added

**FIGURE 4:**
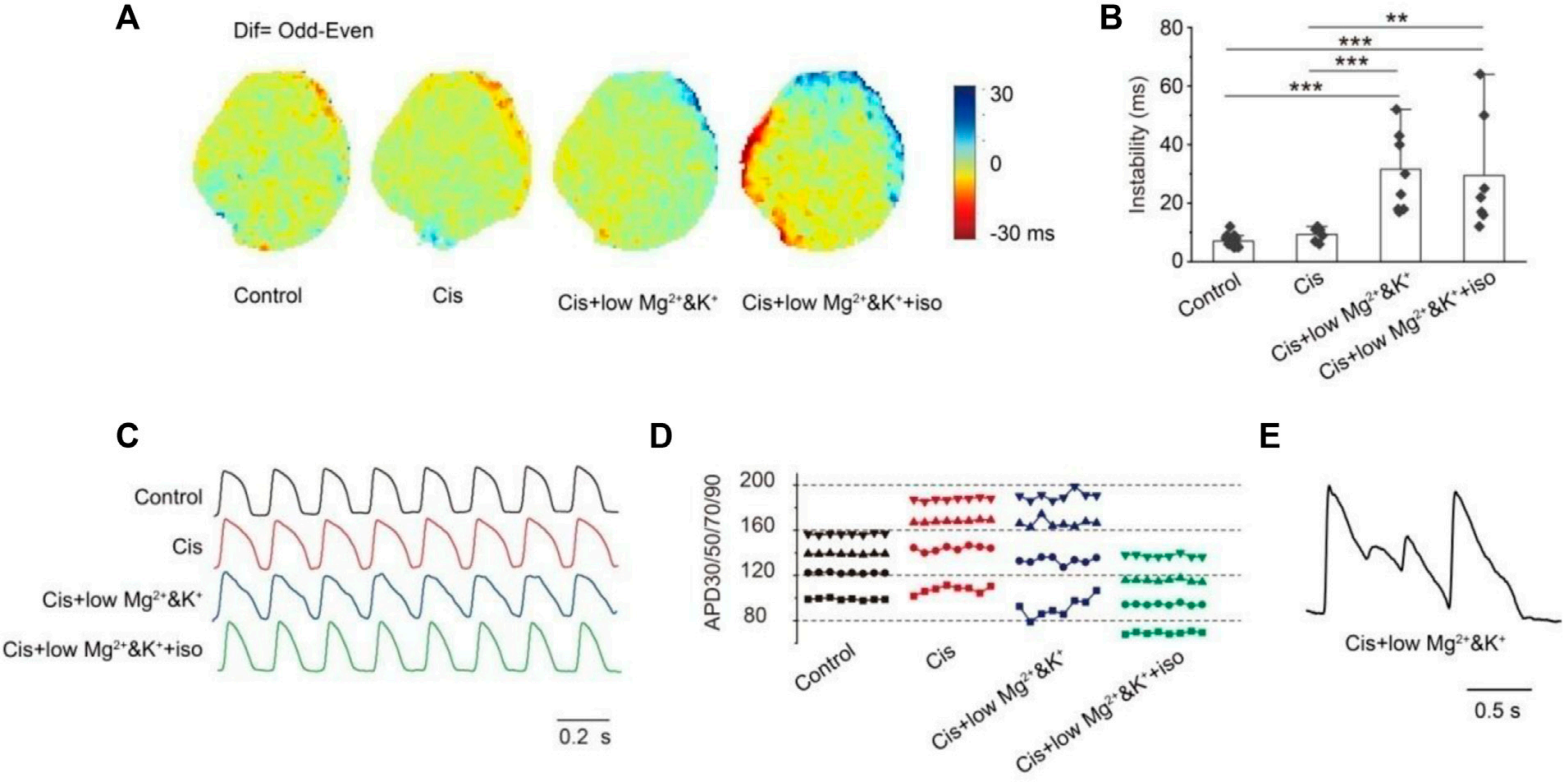
Instability. **(A)** Map of the APD90 difference (Dif) in the epicardial between the odd beats and the even beats under different inducing conditions. Dif = Odd-Even; **(B)** statistics of max dif. There was no significant change in instability when cisapride was given alone, but the instability was significantly altered by the combination of cisapride and low Mg^2+^/K^+^ or that with low Mg^2+^/K^+^ and iso (*p-*values were all less than 0.001); **(C)** action potential representative traces of eight consecutive beats were recorded; **(D)** instability of the action potential duration of eight beats APs in Figure C (■ APD30; ● APD50; ▲ APD70; ▼APD90). APDs fluctuated most significantly after the treatment with cisapride and low Mg^2+^/K^+^;€ EAD occurred only under cisapride combined with low Mg^2+^/K^+^ and iso. **(E)** EAD occurred only under cisapride with low Mg&K and ISO

The instability of consecutive APs provides the substrate for the development of severe arrhythmias. We analyzed the changes in APD instability in the control and in the group with perfusion of 1 μM cisapride alone or combined with lower Mg^2+^/K^+^ or continued combined with isoproterenol. Figure 4A shows the optical maps of APD90 of the odd and even beats under the four conditions tested. As can be seen, it reflects the differences between different beats directly. In this study, the absolute value of the maximum APD90 difference (Max△APD90) between the odd and even beats (Instability = |APD90Odd-APD90Even|Max) was analyzed (Figure 4B). The instability of the APD was similar to that in the control when cisapride was perfused alone (con: 7.12 ± 0.46 ms, *n* = 17 vs. cis: 9.33 ± 0.99 ms, *n* = 6). In the 1 μM cisapride treatment combined with lower Mg^2+^/K^+^ and continued combined with isoproterenol, the APD instability increased significantly (Cis + low Mg^2+^/K^+^: 31.56 ± 4.42 ms, *n* = 9, *p* < 0.001; Cis + low Mg^2+^/K^+^+iso: 19.43 ± 7.45 ms, *n* = 7, *p* < 0.001). Figures 4C,D show the morphology of the recorded consecutive APs and corresponding APD30/50/70/90 with 4 Hz stimulation under the four conditions. As can be seen, the treatment with 1 μM cisapride combined with lower Mg^2+^/K^+^ led to greater instability. In addition, during the experiment, we found that the spontaneous heart rhythm slowed down, and EAD occasionally occurred when cisapride was combined with lower Mg^2+^/K^+^ (Figure 4E).

## Discussion

**FIGURE 5:**
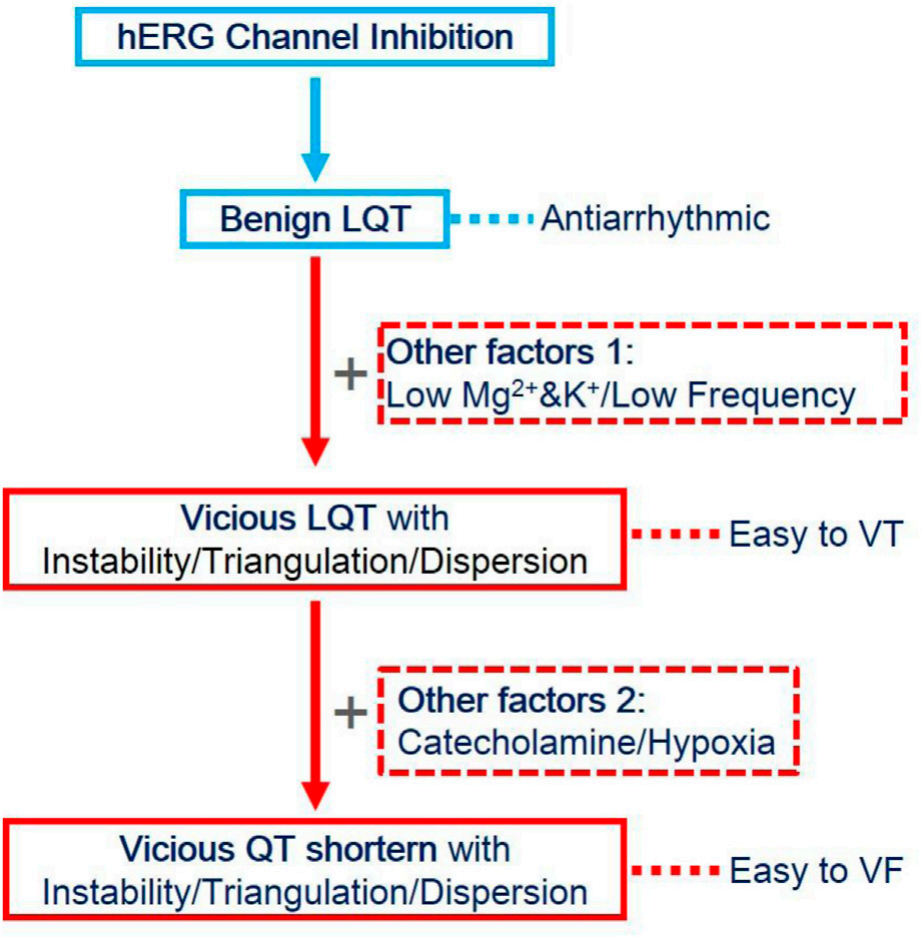
Evolution of QT prolongation caused by hERG inhibition and other factors. HERG inhibition alone led to QT prolongation, along with ERP prolongation, and exerted anti-arrhythmic effects. However, when combined with the effects of low magnesium, low potassium, or low frequency, the prolonged action potential became unstable, triangular, and discrete, and it was easy to induce VT. Still, without the occurrence of VF, VT could not be sustained. When catecholamines or ischemia and hypoxia continued to be combined, the APD was shortened, but instability, triangulation, and dispersion still existed. At this time, VF was easily induced and could be sustained.

In the present study, we established the relationship between hERG channel inhibition alone or combined with other factors. Cisapride dose-dependently prolonged QT/APD and increased the ERP by the inhibition of the hERG channel. Increased ERP is described as a dominant mechanism of the anti-arrhythmic effect (Hondeghem. et al., 2001). When the hERG current was thoroughly inhibited by 1 μM cisapride alone, no EAD/DAD/VT/VF occurred spontaneously or was induced. If LQT exists, ERP begins to increase without instability/triangulation/dispersion and it is less likely to induce arrhythmia, but when LQT exists combined with other factors, such as lower Mg^2+^/K^+^ and low heart rate, it might be to induce arrhythmia and tend to be EAD and VT. Although choosing catecholamine as one of the medicines based on the LQT, the APD was shortened, instability, triangulation, and increased dispersion still existed, which could easily induce VF and persist (Figure 5).

First of all, researchers found that the addition of the action potential is one of the electrophysiological mechanisms for inducing arrhythmias according to a dose-dependent study of cisapride inhibiting the hERG channel and prolonging QT/APD. When hERG currents were completely inhibited by cisapride 1 μM alone, the AP increased without significant changes in dispersion, and no spontaneous or induced EAD/DAD/VT/VF occurred. But when additional experimental factors such as relatively low levels of Mg^2+^/K^+^ and heart rate were considered during the research, the dispersion increased and it is likely to induce arrhythmia (EAD and VT). Second, presently, isoproterenol is recommended as the preferred drug for the treatment of TdP because it could increase the basic heart rate and shorten the QT interval. The research showed that when combined with isoproterenol, the APD was shortened and the dispersion was increased, and it was also easy to induce VF and persistent VT. Inconclusion, isoproterenol also has a risk of causing cardiac arrhythmias.

In 1996, Wysowski and Bacsanyi (1996)reported that LQT and TdP occurred in 57 patients treated with cisapride. Nevertheless, it should be noted that all these patients had combinations with other risk factors, such as coronary disease, arrhythmia (especially atrial fibrillation), renal insufficiency or renal failure, electrolyte imbalance, and long-term intake of medications associated with the prolongation of QT intervals or arrhythmia. In our study, we also confirmed this view that more attention should be paid to other factors in addition to cisapride itself. Here, only the combination of 1 μM cisapride with other factors (such as low magnesium and potassium levels) induced EAD and VT, with a relatively low induction rate.

Isoproterenol is clinically recommended for TdP therapy because it can increase the basal heart rate and shorten the QT interval (Suarez. et al., 2018). However, the pro-arrhythmic risk of isoproterenol has also recently attracted increasing research attention. Wataru Shimizu et al. explained why oproterenol makes the occurrence of TdP easier in acquired or inherited LQT1/LQT2. Isoproterenol lengthens the APD of M cells, shortens the APD of epi and endo cells, and increases the transmural repolarization dispersion of the AP of ventricular myocytes (Shimizu and Antzelevitch, 2000). However, after isoproterenol was administered in our experiment, the APD was shortened, and dispersion decreased while the triangulated form of the action potential became more serious and more likely to induce ventricular fibrillation. Meanwhile, the risks associated with shortening QT with isoproterenol may be even greater than those associated with TdP, which is only 15–20% likely to progress to ventricular fibrillation^[22]^. The cardiac wavelength (conduction velocity * ERP) is an important concept for the development of arrhythmias. Increased *λ* could impede re-entry and vice versa. Shortening QT and triangulation might be the major reason for VF development. Therefore, the clinical application of isoproterenol for TdP therapy should be reexamined.

As for indicators of drug cardiac safety, QT prolongation and hERG inhibition may cause false-positive results, which may result in the blind screening of many valuable drugs (Zang. et al., 2012; Lu. et al., 2019). The findings of our present study show that in addition to hERG inhibition and QT prolongation, combined with changes in instability, triangulation, and dispersion may be a more comprehensive method to evaluate drug cardiotoxicity.

Previous results showed that cisapride (200 μM) caused the largest TDR and induced TdP (2 in 6) in dog left ventricular wedges; however, it should be noted that the stimulation frequency BCL = 2000 ms was far below the normal heart rhythm, and such a frequency only can be induced from epicardial (Di Diego, J. M. et al., 2003b). Differently, we did not observe TdP induction at any concentration in the Langendorff-perfusion whole heart of a guinea pig without the action of other factors. EAD and VT would occur in combination with low magnesium and low potassium, but the probability was low. In our previous experiments, TdP was not induced when E-4031, cisapride, or sotalol were used alone. However, when combined with low magnesium, low potassium, and isoproterenol, VF was easily induced (Shimizu and Antzelevitch, 2000; Di Diego, J. M. et al., 2003b).

## Limitations

First, the ion channel expression of guinea pig ventricular myocytes was different from that of humans, so the experiment on guinea pig heart cannot explain the effect on the human myocardium (Guo. et al., 2009; Horvath. et al., 2020). It has been reported that there is no Ito current in guinea pig cardiomyocytes, and the expression level of hERG is lower than that of humans. Second, cisapride slowed conduction at high concentrations, suggesting that it was not only the hERG channel which was affected by cisapride but other ion channels as well. However, according to *λ* = conduction velocity * ERP, deceleration of the conduction velocity would lead to a higher heart safety risk. Nevertheless, no EAD/DAD/VT/VF occurred spontaneously or was induced by 1 μM cisapride alone. Third, after the addition of isoproterenol, the signal of the edge was not good because of the incomplete stop motivation. Thus, there was a large SE of instability after the perfusion of isoproterenol.

## Data Availability

The original contributions presented in the study are included in the article/Supplementary Material; further inquiries can be directed to the corresponding author.
